# Structural, phylogenetic and docking studies of D-amino acid oxidase activator (*DAOA*), a candidate schizophrenia gene

**DOI:** 10.1186/1742-4682-10-3

**Published:** 2013-01-04

**Authors:** Sheikh Arslan Sehgal, Naureen Aslam Khattak, Asif Mir

**Affiliations:** 1 Department of Bioinformatics and Biotechnology, International Islamic University, H-10 Sector, Islamabad, Pakistan; 2 Institute of Biochemistry and Biotechnology, Department of Biochemistry, Arid Agriculture University Rawalpindi Pakistan, Rawalpindi, Pakistan

**Keywords:** Schizophrenia, Bioinformatics, Modeling, Docking, *DAOA*, Phylogenetic analysis

## Abstract

**Background:**

Schizophrenia is a neurodegenerative disorder that occurs worldwide and can be difficult to diagnose. It is the foremost neurological disorder leading to suicide among patients in both developed and underdeveloped countries. D-amino acid oxidase activator (*DAOA*), also known as G72, is directly implicated in the glutamateric hypothesis of schizophrenia. It activates D-amino acid oxidase, which oxidizes D-serine, leading to modulation of the N-methyl-D-aspartate receptor.

**Methods:**

MODELLER (9v10) was utilized to generate three dimensional structures of the *DAOA* candidate gene. The HOPE server was used for mutational analysis. The Molecular Evolutionary Genetics Analysis (MEGA5) tool was utilized to reconstruct the evolutionary history of the candidate gene *DAOA*. AutoDock was used for protein-ligand docking and Gramm-X and PatchDock for protein-protein docking.

**Results:**

A suitable template (1ZCA) was selected by employing BLASTp on the basis of 33% query coverage, 27% identity and E-value 4.9. The Rampage evaluation tool showed 91.1% favored region, 4.9% allowed region and 4.1% outlier region in *DAOA*. ERRAT demonstrated that the predicted model had a 50.909% quality factor. Mutational analysis of *DAOA* revealed significant effects on hydrogen bonding and correct folding of the DAOA protein, which in turn affect protein conformation. Ciona was inferred as the outgroup. Tetrapods were in their appropriate clusters with bifurcations. Human amino acid sequences are conserved, with chimpanzee and gorilla showing more than 80% homology and bootstrap value based on 1000 replications. Molecular docking analysis was employed to elucidate the binding mode of the reported ligand complex for DAOA. The docking experiment demonstrated that DAOA is involved in major amino acid interactions: the residues that interact most strongly with the ligand C_28_H_28_N_3_O_5_PS_2_ are polar but uncharged (Gln36, Asn38, Thr 122) and non-polar hydrophobic (Ile119, Ser171, Ser21, Ala31). Protein-protein docking simulation demonstrated two ionic bonds and one hydrogen bond involving DAOA. Lys-7 of the receptor protein interacted with Lys-163 and Asp-2037. Tyr-03 interacted with Arg-286 of the ligand protein and formed a hydrogen bond.

**Conclusion:**

The predicted interactions might serve to inhibit the disease-related allele. It is assumed that current bioinformatics methods will contribute significantly to identifying, analyzing and curing schizophrenia. There is an urgent need to develop effective drugs for schizophrenia, and tools for examining candidate genes more accurately and efficiently are required.

## Background

The nature of a human medical disorder is often elucidated through biological markers and behavioral studies. Diagnosis of mental disorders is very difficult because it primarily relies on behavioral markers. An example of a complex mental disorder is schizophrenia (SZ), diagnosis of which depends on abnormal behavior such as paranoia, dampening of emotions and auditory hallucinations. Genome-wide studies have attained a major role in SZ research because high-throughput technologies are valuable for discovering relevant genes. SZ is a psychiatric disorder with severe manifestations - abnormal behavior, disorganized speech and figments of the imagination - and an estimated heritability of about 80% [1]. Negative symptoms can also include affective flattening, avolition, and alogia. Approximately 1% of the population is affected during the course of life. The effects of SZ usually start during the patient’s late teens to early twenties; females have an age of onset five years later than males [2]. A recent meta-data analysis estimated the risk of SZ in males to be about 40% higher than in females [3]. Epidemiological studies of SZ have shown that it occurs in all populations with a prevalence of approximately 1.5-4.5 per thousand and an incidence of 0.17-0.43 per thousand [4].

According to analysis of gene linkage data and meta-analysis of genome scans [5], highly vulnerable genes on chromosomes 1q, 3p, 5q, 6p, 8p, 11q, 14p, 20q and 22q [6,7] contribute to SZ. Both functional and positional candidate SZ genes have been studied and various promising candidates that might be involved in risk for the disease have been identified.

The symptoms of SZ have different dimensions that usually occur together and can reflect substantial variation among patient phenotypes [8-10]. Different researchers have formulated various models of these dimensions but the most widely appreciated 3D models were first proposed by Bilder *et al.* and Liddle [9,11]. These authors concluded that the main symptoms are poverty of speech, formal thought disorder, decreased voluntary movement, psychomotor impairment, bizarre behavior, hallucinations, abnormal acts, inappropriate affects, flat affects, flattening, avolition, and alogia.

A genome-wide association study (GWAS) for SZ was conducted in 2008 but no significant loci were reported, though 7000 samples were used [12,13].

The gene *DAOA*, located on chromosome 13q3, encodes the D-amino acid oxidase activator protein, as shown by functional and expression studies. It is significantly associated with SZ and is also known as G72. The D-amino acid oxidase activator (DAOA) is directly implicated in the glutamateric hypothesis of SZ [14]. When D-amino acid oxidase is activated, D-serine is oxidized and the product modulates the N-methyl-D-aspartate receptor. Modulation of this receptor leads to the cause of SZ; glutamate signaling is involved in important pathways directly linked to SZ [15].

*DAOA* is also involved in other psychotic disorders and can modify the cognitive and negative symptoms of mood. It could be the primary genetic cause of the observed overlap of phenotypes between bipolar disorder and SZ [16].

Bioinformatics has been used for *in silico* analysis of biological queries using mathematical and statistical techniques. X-ray and NMR techniques are expensive and time-consuming for structural modeling of proteins. Screening of small chemical compounds against target receptors by high throughput screening (HTS) is very expensive.

In this work, we predicted the 3D structure and the protein-ligand and protein-protein docking of DAOA using different bioinformatics strategies. The main aim of our research was to predict the 3D structure and docking. The objective of the present study was to elucidate the interactions of DAOA protein with ligands and other proteins and to identify the connection of DAOA to SZ. Protein-protein docking and interaction simulations reveal hydrogen and ionic bonds. The present work was conducted to provide molecular insights into the structure of the protein and to find its most plausible function.

## Results

This paper describes the implementation of an *in silico* technique to recruit and analyze *DAOA,* the most likely candidate gene for SZ. The direct involvement of *DAOA* in disease pathogencity has already been reported in several research studies on SZ.

Initially, a literature search was conducted to explore the most likely candidate gene involved in SZ. A comparative modeling technique (MODELER 9v10) was adopted to predict the three dimensional structure of the protein encoded by the selected gene. The protein data bank (PDB) was checked for the 3D structure of the selected protein, and it was confirmed that no 3D structure had been predicted to date. To check the quality and reliability of the predicted model, the evaluation tools ERRAT and Rampage were used.

Protein-ligand and protein-protein docking of DAOA were simulated. The ZINC and PubChem databases were used to retrieve the ligand and STRING was used to identify protein interactions [17].

*DAOA* has been mapped on chromosome 13, with starting and ending base pairs 06118216 and 10143383 respectively. Homology modeling was implemented to generate the 3D structure of the encoded protein. MODELER 9v10 was used to construct the protein model. A basic local alignment technique (BLAST) was utilized to identify the homology between the target protein and its template. The lowest energy minimization value for the predicted structure was selected for further analysis.

**Figure 1:**
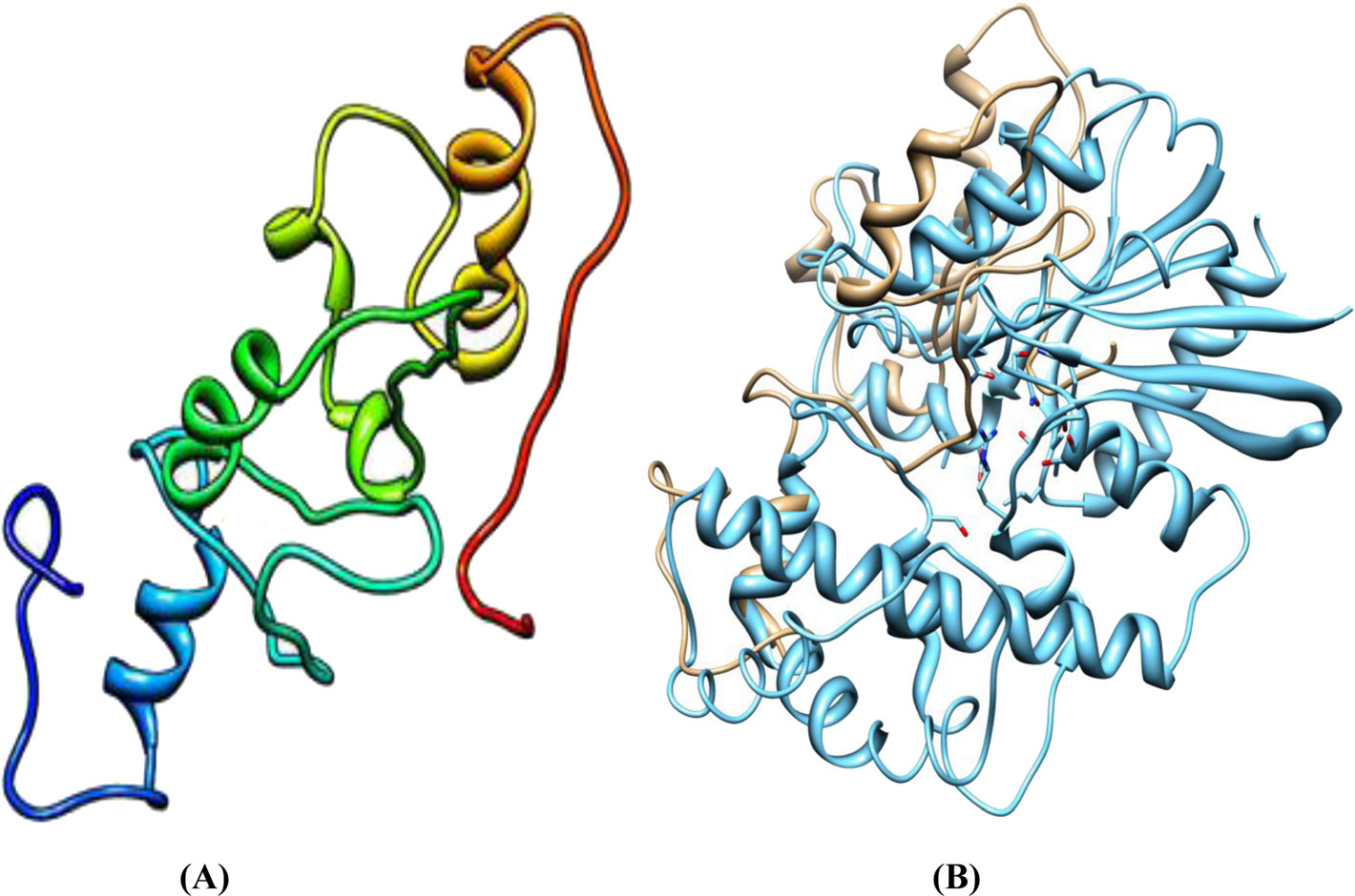
**(A) Predicted structure of DAOA using 1ZCA template with E-Value 4.9 and query coverage 33%.(B)** Structural superimposition of predicted structure and 1ZCA showing high structural similarity. Grey color represents the predicted structure and blue color represents the template used.

**Figure 2:**
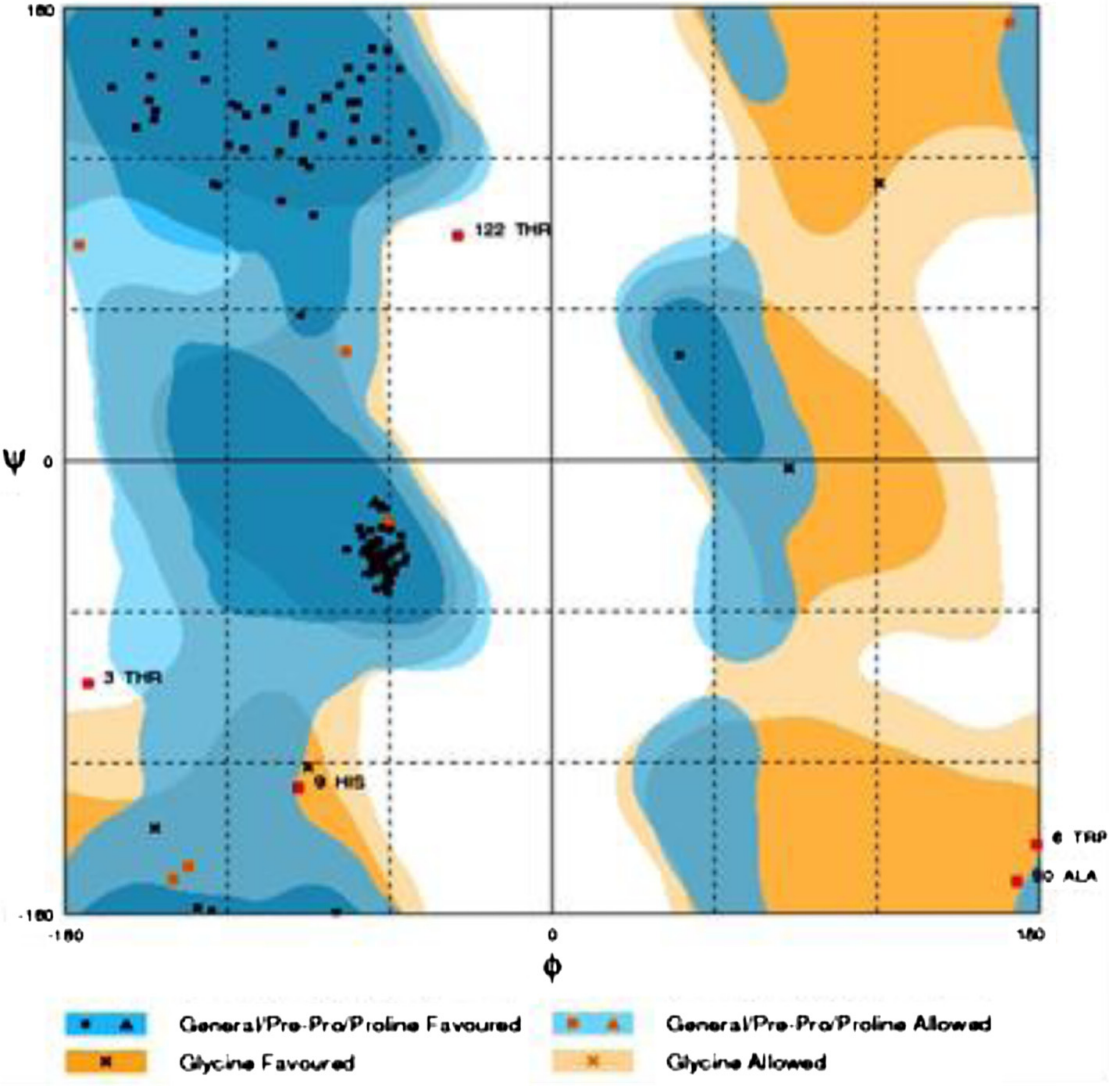
**Rampage defined results in groups of favored, allowed and outlier categories.** Rampage showed 91.1% favored region, 4.9% allowed region and 4.1% outlier region of DAOA.

**Figure 3:**
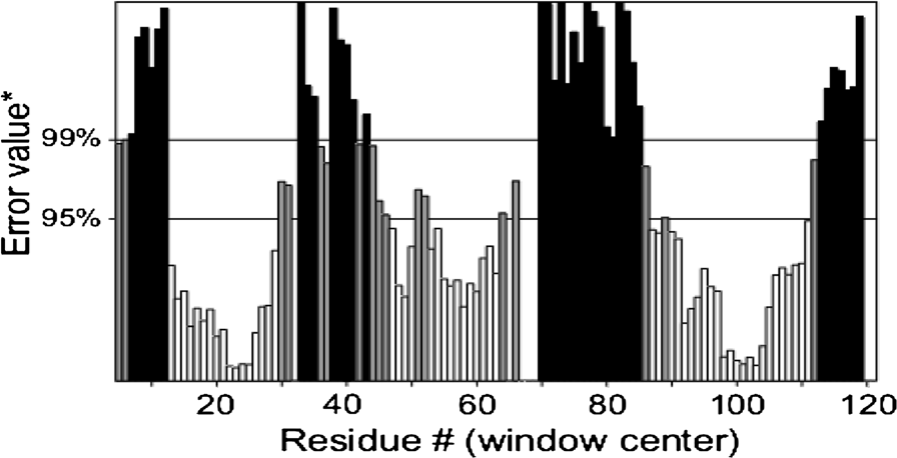
ERRAT gave a quality factor of 50.909% to the DAOA model.

The 3D structure or modeling of DAOA is not known and no structural information can be found for the templates. The amino acid sequence of DAOA in FASTA format was retrieved from Uniprot with accession number A2T115. Table 1 lists the three templates 1ZCA, 1V30 and 2E5K with optimal alignment of the first template and good alignment for the others, sorted by overall quality, query coverage, similarity and E-values. The structure predicted by MODELLER 9v10 with the alpha helices and beta-pleated sheets visualized by Chimera 1.6 is illustrated in Figure 1(A). Figure 1(B) demonstrates a superimposition of structure and template. The predicted structure is evaluated in Figures 2 and 3.

**Table 1:** **Templates for *DAOA *sorted by their overall quality query coverage, identity and E-values**

**Accession ID**	**Total score**	**Query coverage**	**E-value**	**Max-identity**
**1ZCA**	26.9	33%	4.9	27%
**1V30**	30.8	24%	0.16	45%
**2E5K**	26.9	21%	2.5	41%

Mutations in *DAOA* have been reported so text mining was used to retrieve them. The Arg30Lys mutation is directly involved in SZ. Arginine in the wild type protein is replaced with Lysine at position 30, in a highly conserved part of the amino acid sequence. This mis-sense mutation occurs in rare cases but damages the protein when it occurs. The polypeptide backbone is not affected but the side chains of the two amino acids are distinct. Every amino acid has its own specific charge and hydrophobicity value. The mutated and wild type proteins differ in these properties. The mutated amino acid is also smaller than the wild type residue, affecting its interaction with other molecules.

**Figure 4:**
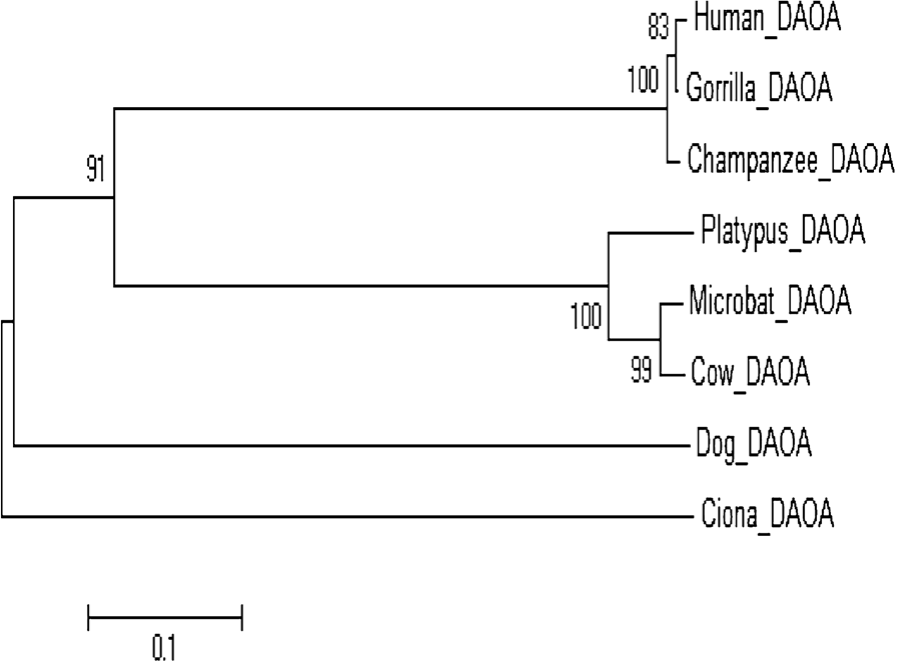
**The evolutionary history of the *DAOA *gene was constructed.** No paralogs of *DAOA* had been reported in biological databases. ENSEMBL Blastp/Blat was also performed for paralogs of *DAOA* but with the same result. Only orthologs of *DAOA* were used to construct the phylogenetic tree. Complete-deletion, p-distance and Uncorrected options were used. Bootstrap values are given as numbers of branches (based on 1000 replications) supporting that branch; values of ≥80% are presented. Amino acid substitution rate per site is shown by the scale bar. Eight orthologs were used and Ciona was the outgroup. Two main clusters were generated. Human, Gorilla and Chimpanzee lie in the same cluster and show sequence similarity. Tetrapods are also closely related to each other in the analysis. Organisms in the same clusters show the highest similarity with point mutations.

A dataset of proteins for a huge range of invertebrate and vertebrate genomes is currently available for analyzing the phylogenetic history of the SZ candidate gene. The phylogenetic neighbor-joining (NJ) tree presented in this study reveals many interesting characteristics of the candidate gene in vertebrates (Figure 4). The phylogenetic history of the gene was analyzed by including the protein sequences from teleosts and tetrapods in the tree; amphioxus sequences were also used as the closest invertebrate relative to vertebrates.

**Figure 5:**
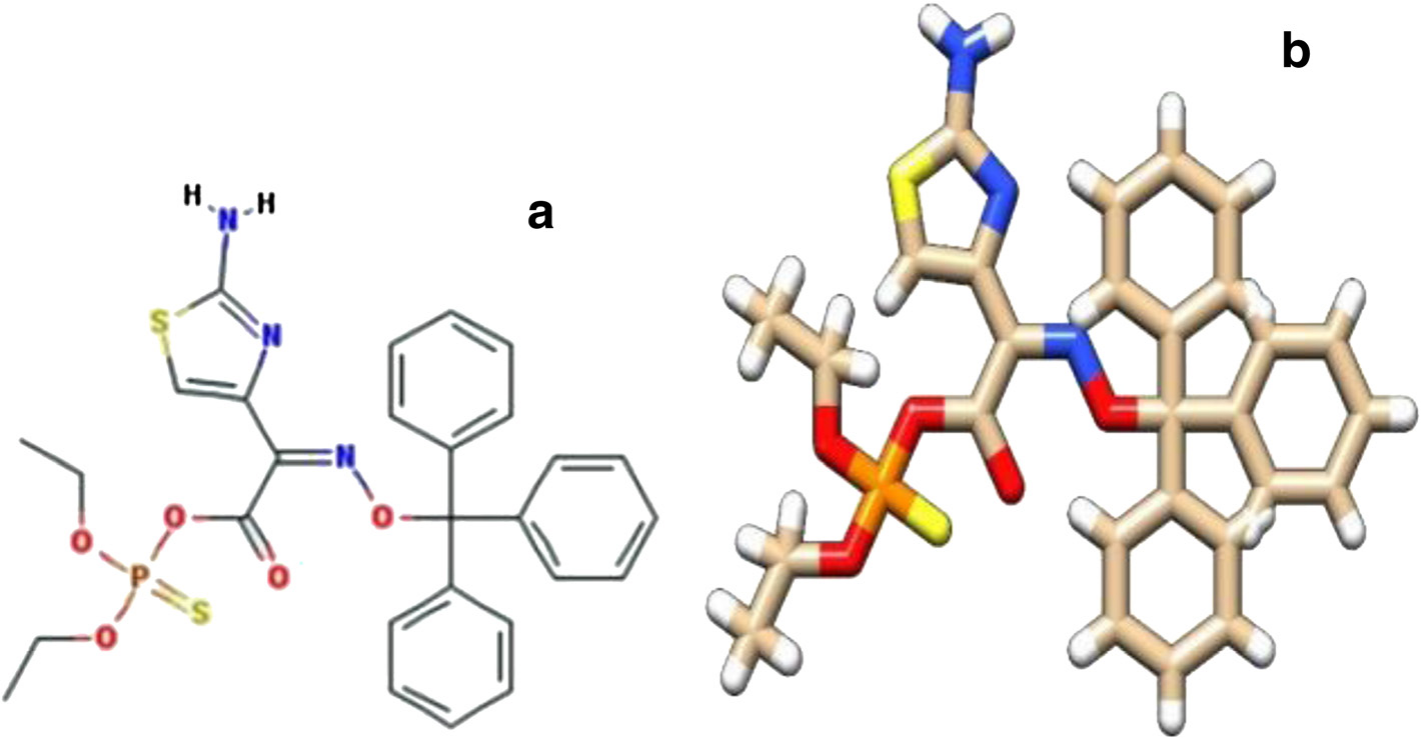
**(a) Structure of ligand (DAOA: O) used for protein-ligand docking with DAOA: molecular formula C**_**28**_**H**_**28**_**N**_**3**_**O**_**5**_**PS**_**2**_**, molecular weight 581.642782.(b)** Structure of O,O-diethylthiophosphoryl (Z)-2-(2-aminothiazol-4-yl)-2-trityloxyiminoacetate drawn using Chimera.

**Figure 6:**
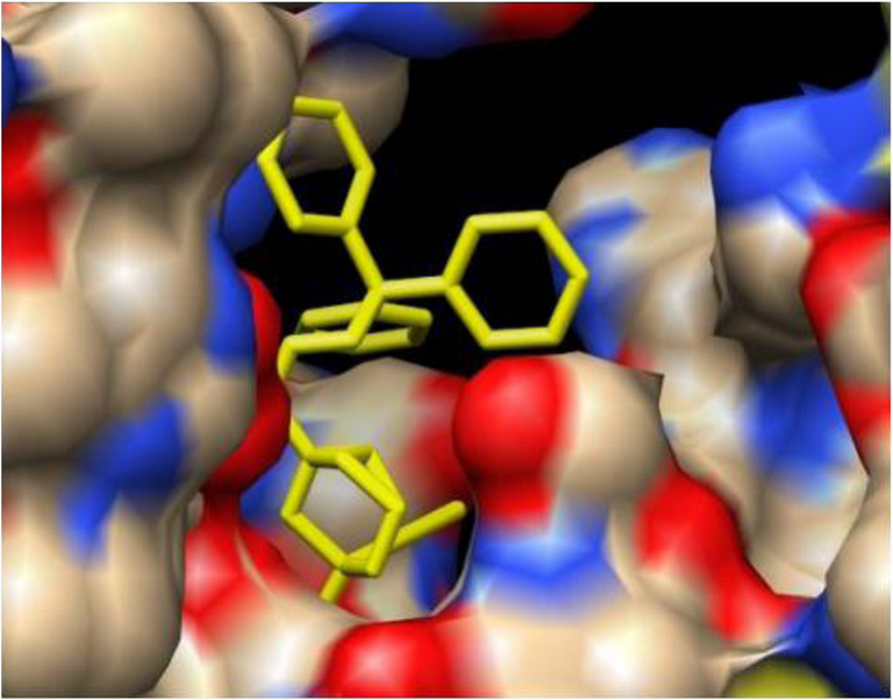
**Frontal view of docked complex of DAOA with O,O-Diethylthiophosphoryl (Z)-2-(2-aminothiazol-4-yl)-2-trityloxyiminoacetate showing ligand in yellow color and stick format.** Receptor protein (DAOA) is shown in surface format.

**Figure 7:**
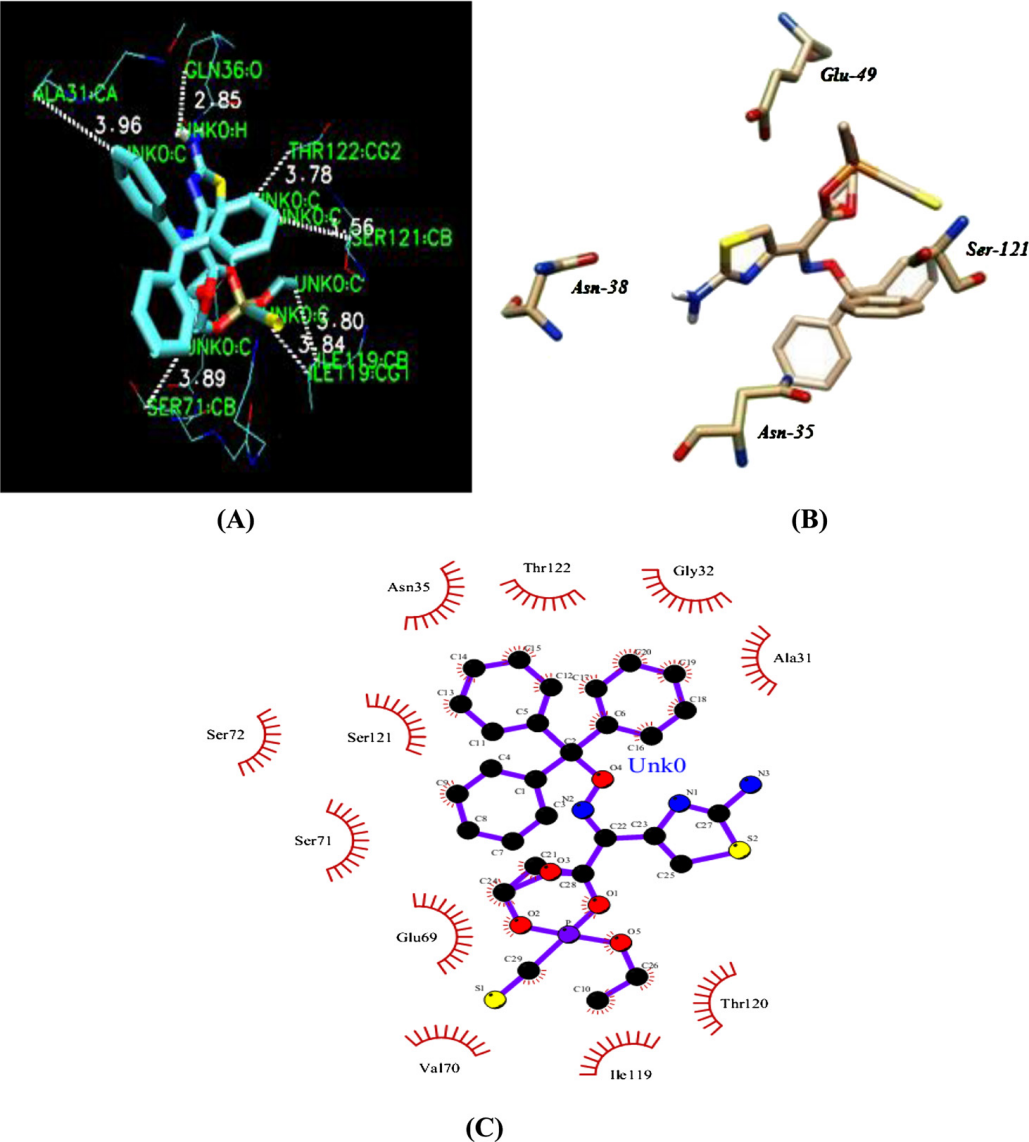
**(A) Amino acid residues of active site of DAOA binding with DAOA: O ligand.** The VMD visualizing tool helped in the visualization and analysis of the protein-ligand docking results. **(B)** Chimera visualization of DAOA and ligand. **(C)** LigPlot analysis of ligand and DAOA.

Autodock VINA docking software was used to investigate how the ligand binds to the respective protein, the binding conformation, functionally interacting residues and best structural information. The ligand retrieved for DAOA is described in Table 2 and its structure is illustrated in Figure 5 (a and b). Docking of different ligand conformations to the protein was simulated. About 50 complexes were generated by the software, and the one with the lowest binding energy was selected for further analysis. The docked complex of DAOA and O,O-diethylthiophosphoryl (Z)-2-(2-aminothiazol-4-yl)-2-trityloxyiminoacetate is illustrated in Figure 6. Figure 7(A) illustrates the docked DAOA complex as revealed by the VMD visualization tool. Figures 7(B) and (C) illustrate the protein-ligand post-docking analysis by Chimera and LigPlot respectively.

**Table 2:** Ligand of DAOA protein was retrieved from the PubChem Ligand database

**Receptor Protein**	**Ligand Database**	**Accession Number**	**Ligand Name**	**Formula**
***DAOA***	PubChem	19894	DAOA:O,O-Diethylthiophosphoryl (Z)-2-(2-aminothiazol-4-yl)-2-trityloxyiminoacetate	C_28_H_28_N_3_O_5_PS_2_

The amino acids present at the active site of the protein were identified by observing the residues within 4Å using VMD visualizing software. The hydrogen, hydrophobic and ionic interactions of the protein-docked ligand complex are described in Table 3.

**Table 3:** Binding interactions of receptor protein DAOA with ligand

**Receptor Protein**	**Hydrogen Bonding O-H**		**Ionic Bonding N-O**	**Hydrophobic Interactions C-C**	
	**Amino Acids**	**Dist**	**Amino Acids**	**Amino Acids**	**Dist**
***DAOA***				Ile 119:CB UNK0:C	3.80
				Ser 71:CB UNK0:C	3.89
	ASN38:OD1 UNK0:H	3.45	No ionic bonds	Ser 121:CB UNK0:C	3.56
	Gln 36: O UNK0:H	2.85		Thr 122:CG2 UNK0:C	3.78
				Ile 119:CG1 UNK0:C	3.84
				Ala 31:CA UNK0:C	3.96

**Figure 8:**
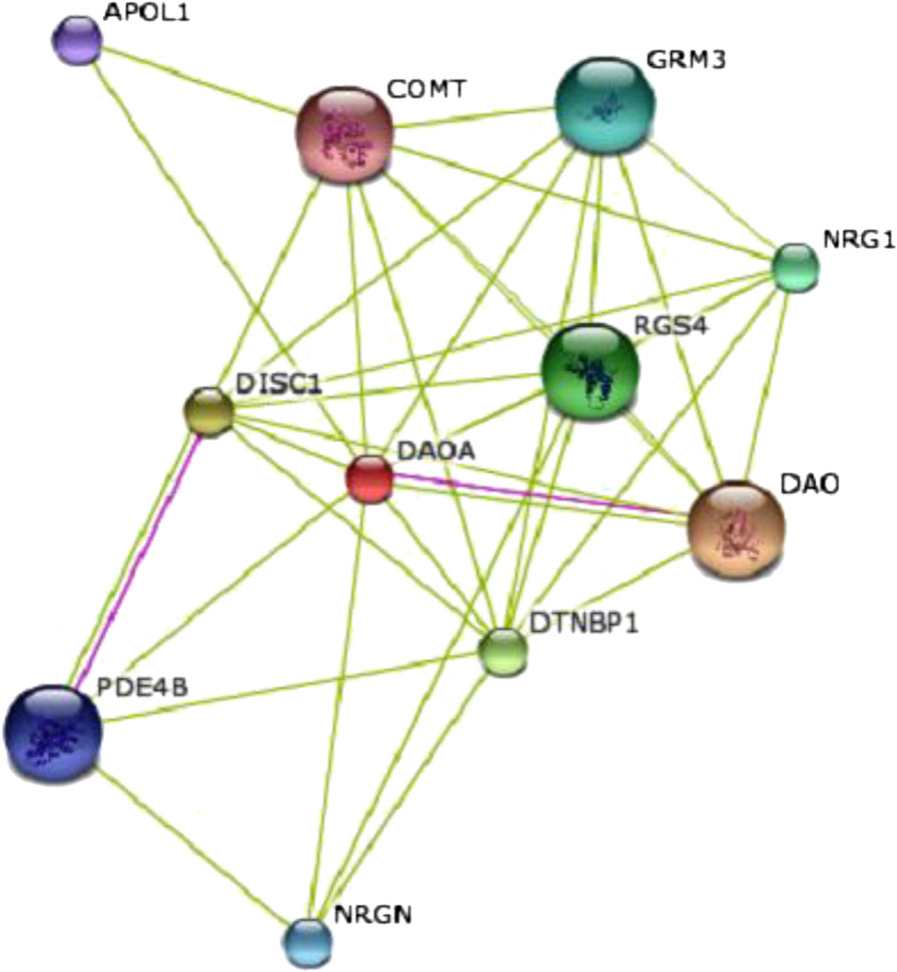
**Interaction network of DAOA showed closely interacting proteins.** DAO protein showed the closest interaction with DAOA with score 0.953; 3D structure is available in PDB.

**Figure 9:**
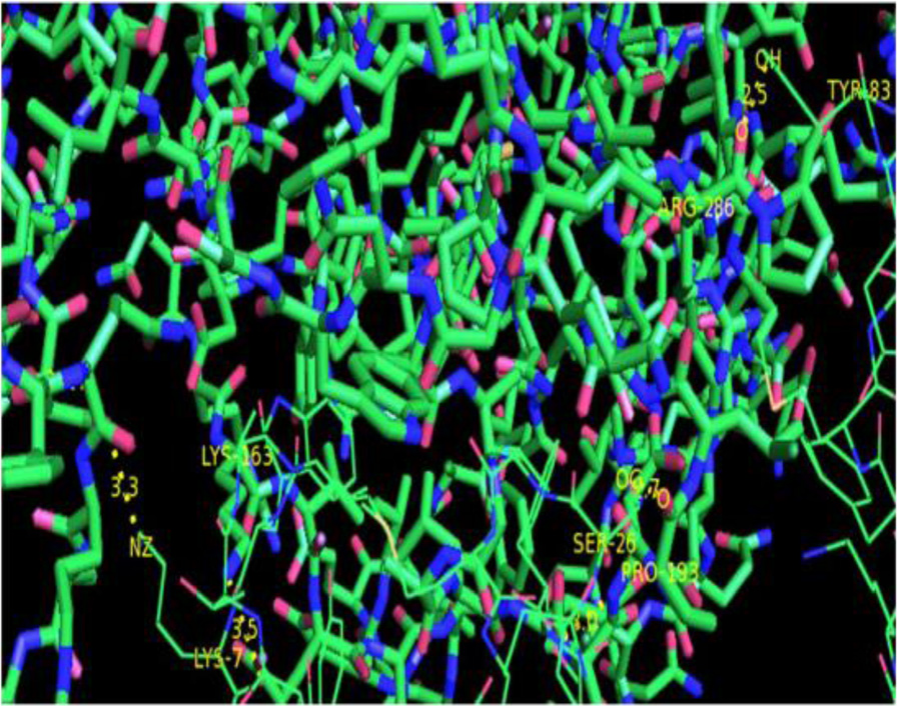
DAOA protein interacted with DAO.

GRAMM-X and PatchDock were utilized to characterize the protein-protein docking of DAOA. The functional interacting partners of DAOA were retrieved from the STRING database. Figure 8 reveals the proteins that interact most closely with DAOA; DAO showed the highest interaction score (0.953). The 3D structure of DAO was retrieved from the PDB with accession number 2E48. The interaction between DAOA and DAO is illustrated in Figure 9. Table 4 lists the interactions and distances between the receptor and ligand proteins.

**Table 4:** Binding interactions for DAOA and DAO

**Receptor Protein**	**Interacting Protein**	**Interactions (Receptor protein → Interacting Protein residue)**	**Bond Distance**	**Interactions Type**
***DAOA***	*DAO*	Tyr-03/OH → Arg-286/O	2.5	Hydrogen Bonding
		Lys-7/O → Lys-163/NZ	3.5	Ionic Bonding
		Lys-7/NZ → Asp-2037/OD1	3.3	Ionic Bonding

## Discussion

SZ is a heterogeneous neural disorder with a spectrum of symptoms such as decrease in volition, displays of antisocial behavior, disordered sense of self, psychomotor slowing and alteration in perception. It has been accepted that SZ is caused by multiple or single dysfunctions within discrete brain regions. Numerous mechanisms and models have been proposed for the development of SZ in terms of the timing, situation and nature of brain changes but the exact mechanisms are still not understood.

No three-dimensional structure of DAOA was reported in the Protein Data Bank or resolved by X-ray crystallography and NMR. MODELLER 9v10 was employed for comparative modeling. Suitable templates for DAOA were identified by running BLASTp, which retrieved three options: 1ZCA, 1V30 and 2E5K. Among these, 1ZCA showed the highest query coverage. The model was predicted by all the templates and 1ZCA showed good evaluation results: 91.1% of the predicted amino acids fell in the favored region and 4.9% in the allowed region. Only four amino acids were outliers. ERRAT revealed a quality factor of 50.909%.

The gene *DAOA* was subjected to mutational analysis and mutations were extracted from biological databases and the literature. Only one mutation of *DAOA* has been reported and authenticated in biological databases: the Arg30Lys mis-sense mutation, which affects protein function because the mutant protein is smaller than wild type and the region around residue 30 is conserved. The mutated protein has abnormal function and its binding and interactions with other proteins are affected, including components of signaling pathways.

A phylogenetic tool (MEGA5) was used to construct a neighbor-joining tree of the selected candidate gene to determine its evolutionary history among human, mammals, tetrapods, primates, birds, teleosts and rodents. *DAOA* has no reported paralogs in biological databases (ENSEMBL and UCSC) or in the literature. Orthologs of *DAOA* were used for tree construction. Ciona was inferred as outgroup in the trees. *DAOA* showed a conserved sequence with primates having few mutations. It has evolved successively from ciona to human.

Protein-ligand docking for DAOA was simulated using AutoDock software. The ligand for receptor- ligand docking was retrieved from different databases, commonly PubChem and ZINC. *DAOA*: O, O-Diethylthiophosphoryl (Z)-2-(2-aminothiazol-4-yl)-2-trityloxyiminoacetate was used for the protein-ligand docking study. The complex with the lowest docked binding energy was selected for post-docking analysis using VMD software (version 1.9.1) and interactions between receptor and ligand were analyzed. In particular, amino acid residues located within 4 Å of the ligand were identified and their prospective interaction types were inferred. Eight interactions were observed between receptor protein and ligand. No ionic interaction was found but there were two hydrogen bonds between an oxygen atom of Asn-38 and a hydrogen in the ligand, and an oxygen of Gln-36 in the receptor protein and a hydrogen in the ligand. The bond distances between Asn-38 and Gln-36 and the ligand were respectively 3.45 Å and 2.85Å. Six hydrophobic interactions were observed between carbon atoms of the receptor protein and ligand. A carbon in Ile-119 interacted hydrophobically with a carbon in the ligand at 3.80 Å bond distance. Another carbon atom of Ile-119 also interacted with a ligand carbon at 3.84 Å bond distance. Ser-121 and Ser-71 of the receptor protein also showed hydrophobic bonding with carbon atoms of the ligand with bond distances 3.56 Å and 3.89 Å respectively. Carbon atoms of Ala-31 and Thr-122 also interacted with the ligand and had 3.96 Å and 3.87 Å bond distances.

GRAMM-X and PatchDock were utilized for DAOA protein-protein docking. The PyMol visualization tool was used for post-docking analysis of protein-protein interactions. DAOA and its functionally interacting partner DAO showed two ionic interactions: an oxygen atom in Arg-286 of the ligand interacted with a hydrogen in Tyr-03 with bond distance 2.5 Å; and a nitrogen atom in Lys-163 of the ligand interacted with an oxygen in Lys-7 with bond distance 3.5 Å. An oxygen atom in Asp-2037 of DAO formed an ionic bond with a nitrogen atom in Lys-7 of DAOA with bond distance 3.3 Å.

## Conclusion

For receptor-ligand interactions, both functional and expressional studies show that the product of *DAOA* interacts with the enzyme D-amino acid oxidase and modulates its activity. Glutamate signaling is involved in important pathways directly implicated in SZ [15]. The ligand examined here (C_28_H_28_N_3_O_5_PS_2_) can be used as a biomolecule, and *in vivo* experiments could be performed in mice to check its effects and interactions, with a view to developing an approved drug for SZ. More than 80% homology between human and primates is strong evidence for an ancestral relationship that will help in predicting protein function and family. Our research suggests a baseline for the design, development and validation of novel drugs against SZ.

## Materials & methods

The amino acid sequence of DAOA (125 residues) was used for homology modeling since it is involved in SZ. The sequence was retrieved in FASTA format from Uniprot Knowledge base with accession number A2T115.

The retrieved amino acid sequence of DAOA was subjected to a protein-protein BLAST (BLASTp) search against the Protein Data Bank (PDB) to identify a suitable template structure for comparative modeling. [PDB ID: 1ZCA] was selected as a suitable template with query sequence having 27% identity, 33% query coverage and E-value 4.9. The automated protein modeling program MODELLER 9v10 was used to generate models. It predicted the 3D structure of the protein by satisfying spatial restraints [18]. The evaluation tools Rampage and ERRAT were applied to assess the predicted 3D model of DAOA. Rampage generated a Ramachandran plot and ERRAT evaluated the quality of the predicted structures.

The mutation was retrieved from the HGMD biological database. The HOPE server was used for mutational analysis. To investigate ancestral relationships, the most popular software for phylogenetic analysis, the Molecular Evolutionary Genetic Algorithm (MEGA 5), was used on *DAOA*. The distance-based approach was applied using Neighbor-Joining, considering a bootstrap value of 1000 replications to construct the phylogenetic tree.

Blind docking was simulated to identify the specific binding site for receptor-ligand and protein-protein interactions. The coordinates of the ligand molecule [Accession number PubChem: 19894] were obtained from PubChem. The SDF (Sql Database File) format of the ligand was transformed into .pdb format using Chem Draw Ultra Version 8.0 [19]. The .pdb formats of protein (receptor) and ligand were used as input files to run AutoDock Vina. To determine the binding affinities between DAOA and the selected ligand, a flexible automated docking program was applied to the active site of the protein using AutoDock Vina. AutoDock has a grid map to aid the actual docking process. The dimensions of the grid were 40 × 40 × 40 points for the ligand with 0.375 Å spacing between the grid points.

The STRING server was used to assess the protein interactions of DAOA. It is an online database of known and predicted protein interactions including direct (physical) and indirect (functional) relationships. Protein docking of DAOA with its interactive protein DAO was simulated using PatchDock and Gramm-X. Visual Molecular Dynamics (VMD) software with a table of all tools utilized was used to visualize assay and post-docking analysis for protein-ligand docking; PyMol software was used for protein-protein docking. The tools employed in this study are listed in Table 5.

**Table 5:** Summary of tools used

**Sr.No.**	**Tools/Database**s	**Output**/**Function**
1	BioGPS	Expression profiling
2	GeneCards	Expression profiling
3	UniProt	Protein Data
4	MODELLER	Structure prediction
5	AutoDock	Docking Analysis
6	HOPE server	Mutational analysis
7	STRING	Protein-Protein Interactions
8	PubChem	Ligand Retrieval
9	ChemDraw	Ligand Drawing
10	GRAMM-X	Protein-Protein Docking
11	ENSEMBL	Retrieval of Phylogenetic Sequences
12	VMD	Binding interactions of docked protein-ligand complexes
13	PyMOL	Binding interactions of docked protein-protein complexes
14	MEGA	Phylogenetic Analysis
15	Chimera	Visualization, Superimposition, Interaction
16	LigPlot	For interaction

## Competing interests

The authors declare that they have no competing interests.

## Authors’ contributions

SAS carried out all the analysis and drafted this manuscript under the guidance of NAK and AM. AM and NAK defined the research theme, designed methods, analyzed the data, and interpreted the results. AM also provided suggestions for interpreting the results. All authors have contributed to, seen, read and approved the manuscript.

## References

[B1] Cardno AG Gottesman II Twin studies of schizophrenia: From bow and arrow concordances to star wars M x and functional genomics Am J Med Genet 2000 97 12 17 10.1002/(SICI)1096-8628(200021)97:1<12::AID-AJMG3>3.0.CO;2-U 10813800

[B2] Gottesman II Shields J Schizophrenia: The Epigenetic Puzzle 1982 New York: Cambridge University Press

[B3] Aleman A Kahn RS Selten JP Sex differences in the risk of schizophrenia: evidence from meta-analysis Arch Gen Psychiatry 2003 60 565 571 10.1001/archpsyc.60.6.565 12796219

[B4] Jablensky A Epidemiology of schizophrenia: the global burden of disease and disability Eur Arch Psychiatry Clin Neurosci 2000 250 274 285 10.1007/s004060070002 11153962

[B5] Liu H Heath SC Sobin C Roos JL Galke BL Blundell ML Genetic variation at the 22q11 PRODH2/DGCR6 locus presents an unusual pattern and increases susceptibility to schizophrenia Proc Natl Acad Sci 2002 99 3717 3722 10.1073/pnas.042700699 11891283 PMC122590

[B6] Harrison PJ Owen MJ Genes for schizophrenia, Recent findings and their pathophysiological implications Lancet 2003 361 417 419 10.1016/S0140-6736(03)12379-3 12573388

[B7] O’Donovan MC Williams NM Owen MJ Recent advances in the genetics of schizophrenia Hum. Psychiatry 2003 8 217 224 10.1093/hmg/ddg302 12952866

[B8] Andreasen NG Arndt S Alliger RJ Miller D Flaum M Symptoms of schizophrenia Arch Gen Psychiatry 1995 52 341 351 10.1001/archpsyc.1995.03950170015003 7726714

[B9] Liddle PF The symptoms of chronic schizophrenia A re-examination of the positive–negative dichotomy Br J Psychiatry 1987 151 145 151 10.1192/bjp.151.2.145 3690102

[B10] Toomey R Wallace C Corrigan P Social processing correlates of nonverbal social perception in schizophrenia Psychiatry 1997 60 292 300 9460098 10.1080/00332747.1997.11024807

[B11] Bilder RM Mukherjee S Rieder RO Pandurangi AK Symptomatic and neuropsychological components of defect states Schizophr Bull 1985 11 409 417 10.1093/schbul/11.3.409 4035304

[B12] O’Donovan MC Craddock N Norton N Williams H Peirce T Moskvina V Nikolov I Hamshere M Carroll L Georgieva L Identification of loci associated with schizophrenia by genome-wide association and follow-up Nat Genet 2008 40 1053 1055 10.1038/ng.201 18677311

[B13] Need AC Ge D Weale ME Maia J Feng S Heinzen EL Shianna KV Yoon W Kasperaviciute D Gennarelli M A genome-wide investigation of SNPs and CNVs in schizophrenia PLoS Genet 2009 5 1000373 10.1371/journal.pgen.1000373 PMC2631150 19197363

[B14] Goff DC Coyle JT The emerging role of glutamate in the pathophysiology and treatment of schizophrenia Am J Psychiatry 2001 158 1367 1377 10.1176/appi.ajp.158.9.1367 11532718

[B15] Carter CJ eIF2B and oligodendrocyte survival: where nature and nurture meet in bipolar disorder and schizophrenia? Schizophr Bull 2007 33 1343 1353 17329232 10.1093/schbul/sbm007 PMC2779884

[B16] Craddock N O’Donovan MC Owen MJ Genes for schizophrenia and bipolar disorder. Implications for psychiatricnosology Schizophr Bull 2006 32 9 16 16319375 10.1093/schbul/sbj033 PMC2632175

[B17] Szklarczyk D Franceschini A Kuhn M Simonovic M Roth A Minguez P Doerks T Stark M Muller J Bork P Jensen JL Mering VC The STRING database in 2011: functional interaction networks of proteins, globally integrated and scored Nucleic Acids Res 2011 39 D561 D568 10.1093/nar/gkq973 21045058 PMC3013807

[B18] Eswar N Eramian D Webb B Shen MY Sali A Protein Structure modeling with MODELLER Methods Mol Biol 2008 426 145 159 10.1007/978-1-60327-058-8_8 18542861

[B19] Mendelsohn LD ChemDraw 8 Ultra: Windows and Macintosh Versions J Chem Inf Comput Sci 2004 44 2225 2226 10.1021/ci040123t

